# What Are the Contextual Enablers and Impacts of Using Digital Technology to Extend Maternal and Child Health Services to Rural Areas? Findings of a Qualitative Study From Nigeria

**DOI:** 10.3389/fgwh.2021.670494

**Published:** 2021-05-14

**Authors:** Bassey Ebenso, Babasola Okusanya, Kehinde Okunade, David Akeju, Adegbenga Ajepe, Godwin O. Akaba, Ramsey M. Yalma, Osasuyi Dirisu, Jamilu Tukur, Manir K. Abdullahi, Okey Okuzu, Matthew J. Allsop

**Affiliations:** ^1^Nuffield Center for International Health and Development, University of Leeds, Leeds, United Kingdom; ^2^Department of Obstetrics and Gynecology, College of Medicine, University of Lagos, Lagos, Nigeria; ^3^Department of Sociology, University of Lagos, Lagos, Nigeria; ^4^Department of Obstetrics and Gynecology, Lagos University Teaching Hospital, Lagos, Nigeria; ^5^Department of Obstetrics and Gynecology, University of Abuja, Abuja, Nigeria; ^6^Department of Community Medicine University of Abuja, Abuja, Nigeria; ^7^Population Council, Abuja, Nigeria; ^8^Aminu Kano Teaching Hospital, Kano, Nigeria; ^9^Instrat Global Health Solutions, Abuja, Nigeria; ^10^Academic Unit of Palliative Care, University of Leeds, Leeds, United Kingdom

**Keywords:** digital health technology, eHealth, video-based training, digitization of healthcare data, maternal and child health, theoretical underpinnings, Nigeria

## Abstract

**Background:** Strengthening health systems to improve access to maternity services remains challenging for Nigeria due partly to weak and irregular in-service training and deficient data management. This paper reports the implementation of digital health tools for video training (VTR) of health workers and digitization of health data at scale, supported by satellite communications (SatCom) technology and existing 3G mobile networks.

**Objective:** To understand whether, and under what circumstances using digital interventions to extend maternal, newborn and child health (MNCH) services to remote areas of Nigeria improved standards of healthcare delivery.

**Methods:** From March 2017 to March 2019, VTR and data digitization interventions were delivered in 126 facilities across three states of Nigeria. Data collection combined documents review with 294 semi-structured interviews of stakeholders across four phases (baseline, midline, endline, and 12-months post-project closedown) to assess acceptability and impacts of digital interventions. Data was analyzed using a framework approach, drawing on a modified Technology Acceptance Model to identify factors that shaped technology adoption and use.

**Results:** Analysis of documents and interview transcripts revealed that a supportive policy environment, and track record of private-public partnerships facilitated adoption of technology. The determinants of technology acceptance among health workers included ease of use, perceived usefulness, and prior familiarity with technology. Perceptions of impact suggested that at the micro (individual) level, repeated engagement with clinical videos increased staff knowledge, motivation and confidence to perform healthcare roles. At meso (organizational) level, better-trained staff felt supported and empowered to provide respectful healthcare and improved management of obstetric complications, triggering increased use of MNCH services. The macro level saw greater use of reliable and accurate data for policymaking.

**Conclusions:** Simultaneous and sustained implementation of VTR and data digitization at scale enabled through SatCom and 3G mobile networks are feasible approaches for supporting improvements in staff confidence and motivation and reported MNCH practices. By identifying mechanisms of impact of digital interventions on micro, meso, and macro levels of the health system, the study extends the evidence base for effectiveness of digital health and theoretical underpinnings to guide further technology use for improving MNCH services in low resource settings.

**Trial Registration:** ISRCTN32105372.

## Introduction

Strengthening health systems to improve access to and quality of primary health care (PHC) services remains challenging for African countries like Nigeria that are characterized by chronic infrastructure deficits, weak and irregular staff training, deficient data management and low health spending (1). Studies show that over 50% of women regard limited financial accessibility as a significant barrier to utilizing skilled maternal, newborn, and child health (MNCH) services (2, 3). These inherent challenges in African countries suggest the need to rethink how health systems are organized and financed. In response, there has been growing global consensus that information and communication technologies (ICTs, also called digital health technologies) can offer innovative ways to strengthen health systems in low- and middle-income countries (LMICs) (4–6). The World Health Organization defines digital health technologies as the practice of employing routine and innovative forms of information and communications technology (ICT) to address health needs (6). The 71st World Health Assembly (WHA) resolution of May 2018 demonstrated this recognition through endorsing digital innovations as a key health system strengthening strategy to help countries achieve targets of universal health coverage (UHC).

Nigeria has implemented several government-led initiatives to assess the role of digital technologies for improving the quality of maternity services. This includes a recent scoping review of ICTs for health implemented across Nigeria (7) which identified over 100 ongoing ICT projects aimed at strengthening diverse health systems functions, with 65 projects targeted at MNCH, 36 projects directed at improving health information systems, 13 projects on training and education of health workforce, and six projects on health financing. This growing interest in ICT projects followed the Federal Government of Nigeria's prioritization of ICTs as a strategy for achieving the targets of a “Saving One Million Lives” initiative (ICT4SOML) implemented to increase access to essential healthcare services for mothers and children (8). In our view, this government-led ICT coordination mechanism deployed through the SOML initiative is an example of national-level institutionalization of digital health (9).

The expanding implementation of ICT projects in the health sector has the potential to improve the cost-effectiveness of care delivery (10) and extend the reach of MNCH services to remote locations. However, the huge cost of providing mobile network infrastructure in rural areas and of constructing road networks to further broaden access to healthcare services has constrained government investment in mobile network infrastructure. Consequently, <55% of the population have access to 3G network coverage (11), thus restricting the chances for using eHealth approaches for healthcare delivery. A promising strategy for addressing such low connectivity and extend the coverage of healthcare services to rural populations is the use of satellite communication (SatCom) with no requirement for investing in infrastructure such as masts and cables (12, 13). However, there is a lack of empirical evaluations of the impact of digital health approaches in general (14), and of SatCom projects implemented in LMICs in particular.

This study addresses this knowledge gap through reporting the evaluation of a digital health project that deployed video training (VTR) of health workers and digitization of health data at scale, supported by SatCom and existing 3G mobile networks. The development of digital health tools has been reported elsewhere (1, 15). This paper aims to report whether and under what circumstances using digital interventions to extend health services to remote areas of Nigeria improved the standard of MNCH services. Secondary objectives were to: (i) identify contextual drivers of acceptability and use of VTR and data digitization interventions, and (ii) assess the impacts of the interventions on the individual, organizational and policy levels of the health system.

To the best of our knowledge, this is the first report on deploying video training and data digitization interventions at scale using SatCom in an LMIC context. This paper defines successful scale in digital technology as the embedding of a digital health product into each level of the health system as opposed to regrading it as a separate activity (9, 16). In this regard, the integration of VTR and data digitization into policy, practices, workflows, and daily lives of health staff in multiple states of Nigeria represents successful scaling of digital technology.

### The Context of MNCH in Nigeria

Nigeria operates a 3-tier healthcare system: primary, secondary, and tertiary. Healthcare provision is an overlapping, concurrent responsibility of the three tiers of government in the country–federal, state, and local government (17). These operational levels have diverse but sometimes overlapping roles and responsibilities. The Federal Ministry of Health (FMOH) develops health policies, provides tertiary healthcare services, and technical support to and regulates the state and local government levels. State Ministries of Health (SMOH) are tasked with providing secondary healthcare services, technical support for and regulation of primary healthcare (PHC) services while Local government authorities implement primary healthcare services (7). The key categories of health workers at the primary health care (PHC) level in Nigeria include doctors, nurse/midwives, community health extension workers (CHEWs) and laboratory staff. However, data is lacking on actual numbers of health workers and salaries of health care workers in the three participating states in this research—Kano, Ondo states, and the Federal Capital Territory. Table 1 indicates that the expected numbers of health workers per 10,000 population in the three states are lower than the national average (highlighted in red). Whilst skilled antenatal care providers and birth attendants exist in most health facilities, they lack essential drugs, and basic tools and equipment to provide high-quality MNCH services. The lack of material resources and dilapidated infrastructure at PHC facilities adversely affect infant and maternal mortality (see Table 1).

**Table 1:** Key PHC indicators by State (in 2018) compared to national average^*^.

**Primary health care indicators**	**FCT**	**Kano state**	**Ondo state**	**National average**
Proportion of physicians per 10,000 people	0.75	0.08	0.71	3.8
Proportion of nurses and midwives per 10,000 people	1.4	0.7	2.3	12
Proportion of CHEWs per 10,000 people	1.43	0.33	0.5	17
Women who received Antenatal care from skilled provider (%)	87.7%	65.3%	92%	67%
Proportion of births delivered in a health facility (%)	63.2%	19.2%	85.6	39%
Proportion of births assisted by skilled personnel (%)	71.6%	21.5%	82%	43%
Women who attended postnatal care within 2 days of birth (%)	61%	23%	49%	42%
Proportion of children age 12–23 months who receive all vaccination (%)	50%	34.3%	39%	31%
Infant mortality rate per 1,000 live births	40	62	49	67
Maternal mortality ratio per 100,000 live births	700	1,549	170	512
Proportion women in paid employment (%)	63	33	56	74%
Proportion of women that participate in household decision-making (%)	56	5	50	44%

* *Nigeria Demographic and Health Survey 2018 (18); WHO health workforce statistics (19). Red, indicator score is worse than national average; green, indicator score is better than the national average*.

Despite the huge reductions in maternal and infant mortality in Nigeria by 56 and 33% respectively since 2000, these indices remain unacceptably high at 512 deaths per 100,000 live births and 67 deaths per 1,000 live births in 2018, respectively (18, 19), with variations in the levels of mortality between regions of the country. The northern states (represented by Kano State) have the highest maternal mortality ratio (with 1,549 deaths per 100,000 live births in 2015) compared to other regions of the country (20).

Table 1 further shows that Kano State has the lowest proportions of: (i) women who received antenatal care from skilled staff, (ii) births delivered in health facilities assisted by skilled personnel, and iii) those who attended postnatal care. Concerning indices of women's empowerment, Kano state has the lowest proportion of women in paid employment and those who participated in household decision-making (33 and 5%, respectively, in 2018).

The above context provided justification for addressing health systems challenges in the three states.

## Materials and Methods

### Study Setting and Design

While the eHealth project aimed to use digital technology to increase the reach and quality of MNCH services in remote areas of Nigeria, the selected states contained both rural and urban centers with variations in the rural-urban divide. Ondo state and the FCT had a 70% rural and 30% urban split while Kano state was more rural with 85% rural and 15% urban split. The three states also differed in terms of religious practices—Christianity and Islam. The population in Ondo State is mostly Christian–65% (21). In the FCT, Christianity and Islam are practiced equally by about 50% of the population, while Islam is widely practiced in Kano–85% (22). We used a mixed-method design to evaluate the acceptability, feasibility, and impacts of implementing novel eHealth tools on MNCH service delivery, as well as understand which contextual factors enabled or constrained implementation of digital interventions in three states of Nigeria. Data collection combined documents review with 279 semi-structured interviews of stakeholders across four phases—at baseline, midline, endline, and legacy evaluation 12-months post-project closedown—to understand: (i) processes and impacts of eHealth tools on standard of MNCH care; and (ii) contextual factors that affected implementation. A legacy evaluation aimed to assess the extent to which project benefits and results were sustained beyond the project's lifetime. As part of the study design, we selected states from different regions of Nigeria (Ondo in west, FCT in central region, and Kano in the north) to facilitate examination of different contextual factors that affected implementation and project outcomes. The eHealth project involved incrementally supplying 126 PHC facilities in intervention local government areas (LGAs) across the three states with tablet computers loaded with data plans to enable the eLearning and health data digitization interventions. Health workers in the 126 facilities were then trained by InStrat (local technology company that implemented the eHealth solutions in Nigeria) staff to use the tablets. Seventy-five of 126 PHC facilities (59.5%) lacked access to 3G mobile network and were supplied with a broadband global area network link-based satellite communication (SatCom) hardware to facilitate internet connectivity in remote rural facilities. The remaining 51 facilities (40.5%) were connected via the regular 3G mobile network and so did not require linking via SatCom.

### Conceptual Frameworks

**Figure 1:**
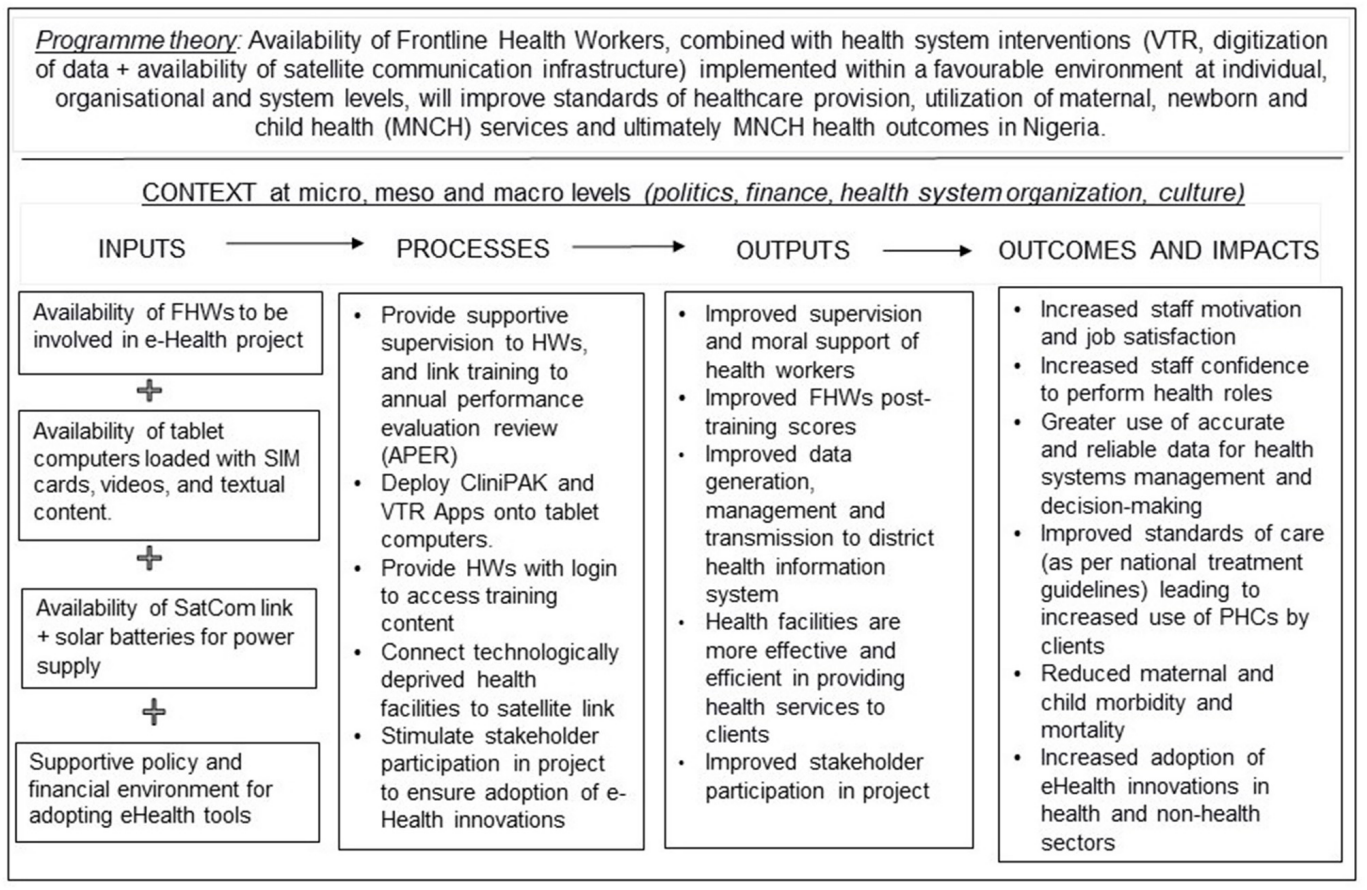
Theory of change model for eHealth project in Nigeria.

The Theory of Change (ToC) model for the eHealth project [see Figure 1], was co-produced with policymakers and project implementers (1), to conceptually evaluate how project inputs and processes created outputs and how outputs subsequently generated outcomes and impacts (23). The ToC also outlines a program theory (see the upper part of Figure 1) of how the project was intended to generate change, along with contextual factors that can influence change.

Key assumptions underlying the project's success, extracted from document reviews and stakeholder discussions, were that:

- Financial support will be secure for the project's 2-year duration (2017–19) and will be extended for another 2 years thereafter (up to 2021)
- Health workers in participating facilities will not be redeployed during the project period.
- Implementing health systems interventions will stimulate technology acceptance and use.

Given the importance of context to achieving project outcomes, we assessed the role of SatCom, VTR Mobile, and data digitization interventions in achieving project results within a wider context (politics, national financial situation, culture, etc.) instead of attributing changes in outcomes to the eHealth project alone. To achieve this, we integrated findings of analysis of documents review with insights from in-depth interviews with stakeholders to understand whether and how the “implementation context” influenced project results, including the influence of competing/concurrent health programs on project effects and outcomes.

To complement the above ToC model, we drew on the modified technology acceptance model (mTAM) to explain stakeholders' acceptance and use of digital health interventions in the three participating states (24). The classic technology acceptance model (25) proposes that two primary factors influence an individual's intention to use a technology: (i) perceived usefulness or the extent to which the technology will enhance job performance, and (ii) perceived ease of use or the extent to which using the technology will be effortless. A person's motivation to use an emerging technology is believed to be higher if that technology is easy to use. In addition to the classic technology acceptance model categories, the mTAM proposes that factors downstream from the technology (e.g., factors within the setting in which a technology is deployed) can shape the perception of usefulness and adoption of a technology (26).

### Study Participants

Three groups of participants were included in this study were: (i) FHWs and facility heads at intervention health facilities; (ii) pregnant women attending the participating facilities, and (iii) policy-makers at LGA and state levels. All participants were at least 18 years of age. Frontline health workers comprised nurses/midwives, laboratory technicians and community health extension workers (CHEWs). Years of experience was not a factor in FHW selection. Within each state, intervention facilities were purposively selected across LGAs. The 126 health facilities included in this study are sub-categorized following the National Primary Health Care Development Agency criteria as Primary Health Care (PHC) facilities, Comprehensive Health Centers (CHCs), Health Posts, and Basic Health Centers, and were unequally distributed in number and type across the three study states (Table 2).

**Table 2:** Distribution of participating health facilities by their locations in Nigeria.

**Participating states**	**Region**	**Population (2006 census figures) [17]**	**Participating local government areas**	**Facility type distribution by state**				
				**Primary health center**	**Comprehensive health center**	**Health post**	**Basic health center**	**Total**
Ondo state	Western Nigeria	3,460,877 (18th/37)	Akoko South, Idanre, and Odigbo	58	4	0	0	62
Kano state	Northern Nigeria	9,401,288 (1st/37)	Dawakin Tofa and Sumaila	7	0	21	7	35
Federal Capital Territory	Central Nigeria	1,406,239 (37th/37)	Gwagwalada	26	2	1	0	29
Total facilities				91	6	22	7	126

PHC facilities, Health Posts and Basic Health Centers all provide primary-level care and serve as the first point of contact for service users, whereas Comprehensive Health Centers provide secondary level care and serve as referral centers for PHC facilities, health posts and basic health centers. Primary level facilities are mostly staffed by community health extension workers (CHEWs), but no midwives or medical doctors, while secondary-level facilities are staffed by CHEWs, medical doctors, nurses, and midwives. In Nigeria, CHEWs are usually trained for 2–3 years in the Schools of Health Technology to provide basic public health services at primary level facilities as well as assist nurses and midwives in their duties [18]. The eHealth interventions reported in this paper targeted CHEWs who are found in all four types of health facilities, and nurses/midwives who are only found in PHC facilities and Comprehensive Health Centers. In Health Posts and Basic Health Centers, there was typically one FHW available for the study (often the facility manager), whereas PHC facilities and Comprehensive Health Centers had at least two FHWs available, as they are usually staffed by a mixed cadre of staff.

Members of the research team recruited all selected FHWs after receiving the facility manager's permission, explaining the objectives of the study to FHWs and obtaining their consent to participate (see Supplementary Material 1), followed by an orientation and training to prepare FHWs to embrace and use tablet computers and the eHealth interventions. Beyond training health workers to use tablet computers, the InStrat staff and members of the research team provided ongoing technical and programmatic support, respectively, to ensure that tablet computers and the SatCom infrastructure continued to function and that FHWs remained engaged to using eHealth tools in spite of potential attrition. Health facility managers specifically helped to ensure that trained staff were not redeployed to non-participating sites during the period of the project.

### eHealth Interventions

The digital health interventions involved providing 126 facilities with a tablet computer containing a video training application (VTR Mobile) and a data digitization application.

It is important to clarify contextual factors that informed the disparity in the population size of participating states and the number of primary health facilities included in the study (Table 2). Kano State, the largest state with over 9 million people had the lowest number of primary health centers (i.e., 7). Ondo state with ~3.5 million population had 58 primary health centers. In the FCT with ~1.5 million population, all the 24 primary health centers available in the intervention LGA were included in the study. The driving factor behind the very low numbers of primary health centers in Kano is related to volatile security situation. Frequent kidnapping and armed banditry in Kano led to a decision by the wider eHealth program to reduce the exposure of research personnel to security risks by reducing the numbers of primary health centers in areas of Kano affected by the insecurity. Ondo state and the FCT were comparatively more stable in terms of security. The numbers of health posts (*n* = 21) and basic health centers (*n* = 7) in Kano were thus increased to enable researchers to gather perspectives from staff at the primary health facility level, leading to the largest representation of other primary health facilities in Kano State.

#### VTR Mobile Application

Enabled users to access video, audio, and text-based MNCH materials through the internet. The eLearning videos used in our study were developed by *Medical Aid Films* (27) and the *Global Health Media Project* (28) and accessed via the *ORB Platform* (29), which was developed by *mPowering Frontline Health Workers Partnership* (30). The ORB platform hosts high quality medical content that can be used under a Creative Commons License to train front line workers via the internet or downloads to mobile devices. The educational videos provided clear educational content and engaging clinical scenarios focused on MNCH care, specifically antenatal care, basic obstetric care, perinatal care, and postnatal care. The contents of the videos were selected in consultation with the relevant state Ministries of Health. The videos were delivered to users via the structured VTR Mobile program.

#### Data Digitization Application

Was a tablet computer-enabled point-of-care data capture and decision support tool that enabled users to capture patient-level health information and send appropriate data to remote servers through mobile networks. The software provided an electronic medical record (EMR) that incorporates data on patient registration, demography, vital signs, clinical diagnosis, treatment, case review, and administrative task support. The software triggers immediate alerts for at-risk patients, and referrals to secondary health systems and on-demand reporting to enable health administrators to increase productivity and improve the patient clinical experience. The software and content were developed and is owned by Vecna Cares Charitable Trust, Cambridge, Massachusetts, USA. The application uses locally available technologies and infrastructure to transfer medical records to remote computer servers while making data available for decision support and project management.

The staff of the e-Health interventions provider [InStrat Global Health Solutions] created user logins and provided the same to study participants to enable them to log into and use the VTR Mobile and data digitization Applications, which also tracked usage of the Apps.

### Data Collection

We used a combination of document reviews and semi-structured qualitative interviews with 98 service users, 63 health workers, 90 facility managers, and 43 policymakers across 4 time periods (at baseline, midline, end-line, and 12 months post-project closedown) to assess the acceptability, feasibility and impact of digital interventions. Purposive sampling was used to ensure that all four groups (service users, health staff, facility managers, and policymakers) were represented in interviews. Interviews were conducted by two social scientists [DA, MKA] and five medical doctors [KO, JT, OD, RMY, and GOA], trained in qualitative interviewing and the use of the most significant change techniques. Data collectors provided potential participants with the study information sheet to explain study objectives and help participants decide whether (or not) to partake in the study. Participants were given at least 24 h to express an interest in participating in the study. Interview guides were pre-tested before they were administered on the field. See Supplementary Material 2 for an example of an interview guide. Interviews lasted 20–30 min and were conducted in a private setting in a health facility, audio- recorded, transcribed verbatim, and where appropriate translated into English for analysis. Documents reviewed as part of data collection included: reproductive health policies, ICT policies at national and state levels, and reports of State MNCH programs identified through discussions with program implementers and examined to understand how the eHealth project was meant to work and the assumptions held by stakeholders. Table 3 shows the key features of each phase of the study and data collection methods adopted during the phase.

**Table 3:** Features of and methods adopted for data collection during phases of study.

**Phase and dates of evaluation**	**Features of phase**	**Methods of data collection**
**Baseline** 23 May to 30 June 2017	a) Developed a working theory of how the project is intended to function. b) Ascertained status of key performance indicators before implementation of project c) Ascertained how PHC facilities generated and transmitted health data to the national health management information system (NHMIS) d) Ascertained service users' motivation for using PHC facilities	a) Review of ICT and reproductive health policies b) Literature review of effectiveness of digital health tools c) Literature review of models of technology acceptance d) Semi-structured interviews with 73 stakeholders across three states: - Service users (*n* = 31) - Health workers (*n* = 0) - Facility managers (*n* = 31) - Policymakers (*n* = 11)^*^
**Midline** 19 February to 9 March 2018	a) Compared performance of KPIs with baseline b) Assessed acceptability and effectiveness of digital health interventions	Semi-structured interviews with 84 stakeholders across three states: - Service users (*n* = 24) - Health workers (*n* = 24) - Facility managers (*n* = 24) - Policymakers (*n* = 12)
**Endline** 02–11 January 2019	a) Compared performance of KPIs with baseline b) Assessed acceptability and effectiveness of digital health interventions and c) Assessed extent of achievement of health outcomes	Semi-structured interviews with 90 stakeholders across three states: - Service users (*n* = 27) - Health workers (*n* = 27) - Facility managers (*n* = 23) - Policymakers (*n* = 13)
**Legacy evaluation** 02–19 March 2020	a) Compared performance of KPIs with baseline b) Assessed the extent of longevity of achieved project outcomes c) Assessed the impact of digital health tools	Most significant change interviews with 47 stakeholders in two of three states (Kano state was dropped due to budget constraint): - Service users and community members (*n* = 16) - Health workers (*n* = 12) - Facility managers (*n* = 12) - Policymakers (*n* = 7)

* *Policymakers with oversight for ICT and/or MNCH were interviewed at Local Government Area and state levels*.

### Data Analysis

Data analyses during baseline, midline, end-line and legacy evaluations were conducted by seven authors who conducted interviews across all stakeholder groups (KO and DA for Ondo state; OD, GOA and RMY for the Federal Capital Territory; and MKA and JT for Kano State). Following analyses at the baseline phase of the study, the researchers produced state-level reports that formed the dataset for the country-level evaluation report for each phase (written by BE and MA and approved by all authors) to make sense of the effectiveness and impacts of eHealth interventions and contextual factors that influenced project results. Framework approach was used for data analysis while allowing for the emergence of new themes. Framework analysis involves the stages of familiarization with data, coding, indexing, charting, mapping and interpretation (31). Analysis was conducted manually. The seven authors read and re-read the transcripts for each state to be thoroughly immersed in the data. Six transcripts (representing the four stakeholder groups) were then randomly selected across the three states, from where a coding frame was created and defined by DA, OD, and BE, who met virtually (via *Zoom*) to compare similarities and differences and to agree on which categories to use in further coding of data. The process was then repeated till all remaining transcripts (for FHWs, facility managers, patients, and policymakers) were coded, necessitating expansion of the coding framework with new categories to accommodate data from stakeholder transcripts. Additional to the above process of inductive coding, we used the mTAM model as a thematic framework to guide deductive analysis by moving from codes to themes to identify individual, organizational and wider contextual drivers, and/or barriers of acceptance and use of technology by FHWs. The findings reported in this paper describes the responses of study participants using the themes and subthemes identified. Our reporting aligns with the Consolidated criteria for reporting qualitative research—COREQ (32).

### Ethics Approval

Approval for the study was granted by the University of Leeds School of Medicine Research Ethics Committee (MREC16-178), the Ondo State Government Ministry of Health (AD.4693 Vol. II/109), the Kano State Ministry of Health (MOH/Off/797/T1/350) and the Federal Capital Health Research Ethics Committee (FHREC/2017/01/42/12-05-17).

## Results

### Characteristics of Stakeholders Interviewed

A total of 294 stakeholders were interviewed over the four phases of data collection for this study, 98 of whom (or 33% of stakeholders) were pregnant women and new mothers attending healthcare facilities for MNCH services; 63 people (or 21.4% of stakeholders) worked at health facilities as nurse/midwives and CHEWs. Ninety people (or 30.6% stakeholders) were facility heads while the remaining 43 people (or 14.6% of stakeholders) were policymakers. The first three stakeholder groups were interviewed at the facility level while policymakers were interviewed at LGA, state and national levels. See Table 4 for details of stakeholder groups interviewed by phase of study.

**Table 4:** Respondents interviewed by stakeholder group and phase of data collection.

**Respondent group**	**Baseline**	**Midline**	**End-line**	**Legacy evaluation**	**Total (%)**
Service users	31	24	27	16	98 (33%)
Health workers	0	24	27	12	63 (21.4%)
Facility managers	31	24	23	12	90 (30.6%)
Policymakers	11	12	13	7	43 (14.6%)
Total	73 (24.8%)	84 (28.6%)	90 (30.6%)	47 (16%)	294 (100%)

### Main Themes

Five main themes emerged from the analysis of documents and interview transcripts: (1) contextual enablers of technology adoption by governments, (2) drivers of technology acceptance, (3) barriers to the use of technology, (4) effectiveness and impacts of digital interventions, and (5) increased adoption of eHealth innovations in the health sector.

#### Contextual Enablers of Technology Adoption by State Governments

Two contextual enablers of technology adoption by state governments in Nigeria are: (i) a supportive policy environment and (ii) a private-public partnership approach to developing and delivering digital innovation. Concerning policy context, documents analysis showed that the Federal Government of Nigeria adopted ICTs in 2013 as a priority strategy for achieving targets of the “Save One Million Lives” (SOML) initiative (33) that aimed to broaden access to essential PHC services for vulnerable mothers and infants. In support of the SOML initiative, state governments across Nigeria deployed several ICT apps including eLearning and data digitization apps to improve the quality of MNCH.

Analysis further showed that the three participating states of Kano, Ondo, and the FCT had verifiable ICT policies at the time of endline assessment (March 2019), compared to baseline assessment (June 2017) when only Kano state had an ICT policy. Kano state was the first state in Nigeria to develop an ICT policy (in 2009), whereas Ondo state and the FCT launched their ICT policies in 2017 and 2018, respectively. The supportive policy context in the states facilitated the adoption of digital technologies (VTR, data digitization, and SatCom) mainly because the objectives of the eHealth project were aligned to the Federal Government's commitment to achieving targets of the “Save One Million Lives” initiative. The benefits of implementing digital technology in the healthcare sector influenced the governments of Ondo State and the FCT to develop and launch their ICT projects.

Regarding private-public partnerships (34), analysis revealed that this eHealth project was a promising example of public-private partnerships (PPP) between local technology companies and the governments in Nigeria for designing and delivering digital health initiatives for public benefit. The PPP approach provided a viable chance of success for sharing resources, capabilities and project risks. In this case, the local technology company provided funding, ICT capability and digital health solutions including hardware, software and content, while State Governments provided healthcare infrastructure and the population reach to make the project a success. The implementing company for the project (InStrat Global Health Solutions) has over 7 years of PPP involvement with State and Federal Governments to improve innovation and efficiency in the generation and performance of healthcare services in Nigeria. This ICT-focused PPP started during the Ebola Crisis of 2015/16 when InStrat supported the Ondo State Government to develop and deploy an Ebola-related eLearning App for training frontline health workers (35), which involved initial financial inputs, technical know-how, and operational efficiency. The successful testing of the Ebola eLearning App in Ondo state led to the adoption of VTR and data digitization interventions in the three states of Kano, Ondo, and FCT.

#### Drivers of Technology Acceptance Among Health Workers

Semi-structured interviews with FHWs, facility managers and policymakers demonstrated wide acceptance of video training and data digitization interventions as essential tools for improving quality of training, data management and standard of healthcare delivery.

Consistent with the classic technology acceptance models, stakeholders cited two principal drivers of technology acceptance: (a) perceived ease of use of Apps and digital platform, and (b) perceived usefulness of video training and data digitization Apps for improving individual performance and facility-level decision-making, respectively. However, given the significance of contextual factors in shaping technology acceptance, participants also described four organizational and two wider contextual factors downstream from the technology that shaped the use of VTR and data digitization tools. Organizational factors described were (a) accessibility to tablet computers in workplace, (b) prior training, (c) technical and programmatic support throughout the duration of the project, and (d) workload issues in health facilities. Two wider contextual factors were poor internet access and the lack of mains electricity supply in remote areas.

It was common for stakeholders across the three states to cite multiple drivers of technology acceptance (with some mixing technology-related and contextual factors) in their responses. Some of the quotes below therefore refer to more than one determinant of technology acceptance and use in the same narrative.

##### Perceived Ease of Use Is Associated With Access to Technology

Health workers and facility heads found it easy to use technology. They reported how access to tablet computers in health facilities facilitated acceptability and use of both VTR Mobile and data digitization Apps. Regarding VTR mobile, a health worker claimed:

“Seriously the videos [on tablets in the facility] are helping us to carry out our task, because we no longer call on FHWs from other facilities…we don't need many complex equipment. These videos just put us through [guide us] with what procedure we want…It is saving lives of women and infants and our own lives too. The clients are now having more confidence in us” (*FHW in Gwagwalada, FCT*).

This is supported by another FHW who narrates the ease of using VTR for improving staff knowledge compared to face-to-face training:

“The use of video training is more beneficial than the face-to-face training because we use it together with all the staff in this facility. The use of the video is easier to us to update our knowledge than face–to-face training” (*FHW in Dawakin Tofa, Kano State*).

In the next quote, a policymaker summarizes how the ease of understanding of the data digitization App shaped perceived ease of use of the technology by health workers irrespective of their level of education.

“The successes in the eHealth project are first, the ease of data collection and second, they [health workers] don't need to be experts in terms of knowledge [or training] to use the App to capture data. That is the main difference between this tablet [supplied by the eHealth project] and other tablets [supplied by competing health programs]. That is because, for the other tablets, you will have to go through a lot of processes, but this one [supplied by the eHealth project] is strictly for data collection. So, I think the most important part is the ease of use. In other words, somebody that is not a health management officer is able to use the tablet supplied by the eHealth project. Even our *Ad hoc* staff are using it to collect health data now, provided they know how to read, count numbers and count figures. I think those are the two most critical successes” (*Policy Maker, Ondo State*).

For this policymaker, the remarkable user-friendliness of the digital devices and Apps (when compared to devices supplied by competing health programs) increased acceptance and usability by non-experts and *Ad hoc* health workers.

Whereas the last two quotes emphasized the importance of accessibility to and user-friendliness of technology in shaping perceived ease of use of technology, it is equally important to stress that acceptance and use of digital technology were also shaped by: (i) prior training of FHWs provided at the beginning of the project to increase familiarity with technology and (ii) ongoing support to solve technical problems (see section Study Participants).

##### Perceived Usefulness of Technology for Improving Individual Performance

A central driver of acceptability reported by FHWs across the three states was the usefulness of VTR Mobile for improving the quality of health care provision. Participant narratives illustrate the utility of computer-optimized videos for improving staff knowledge, boosting their capability to manage birth-related complications and prevent avoidable deaths in pregnant women and their infants.

“You know sometimes when you don't know something [about a topic], and when you continue to receive cases related to that topic, you will be discouraged and doubtful, because you don't know what to do. But when you already have the solution in your hands [as is the case with VTR], then, you will be confident in what you do, and in the answers, you provide to your clients” (*FHW in Onikokodiya, Ondo state*).

Although we had expected the introduction of VTR Mobile to increase staff knowledge and confidence to provide life-saving care, an unexpected finding was the resourcefulness of FHWs to integrate video viewing sessions into patient health education slots during ante-natal (ANC) clinics. This unintended but creative use of clinical videos, in turn, inspired pregnant women to tell others about new technologies at the study sites, leading to increased patient attendance at ANC clinics—a sign of positive care-seeking behavior. Two service users corroborated the use and usefulness of videos for improving the quality of care:

“There was a video that they [the eHealth project] brought here that most of the health workers watch during the project. The videos show how to manage and how to even prevent bleeding during pregnancy. When you cannot prevent it, it shows you what you can do to manage it…since they started watching it, that kind of bleeding is not a problem here anymore” (*Service User, in Gwagwalada, FCT*).

“I have witnessed the staff using a new equipment [i.e., computer tablet] most of the times I visit the facility. I used to see him [the FHW] touching something that looks like a big handset when he is seeing patients, but I cannot really say what they use it for” (*Service user, in Kano state*).

The above quotes illustrate the variation in service users' awareness of the introduction and use of technology across states depending on their regional location. Service users in Kano state were seemingly uncertain of why FHWs used tablet computers while their peers in the FCT understood that FHWs watched clinical videos to guide and/or improve their practice.

The next two quotes explain why service users in other regions of Nigeria understood the implemented digital technology by capturing how watching clinical videos motivated pregnant women in the FCT to attend ANC classes.

“Before this project, for someone like me, I wasn't that active in coming to this place [i.e., ANC classes] but when I came for the first time 6 months ago, I found it encouraging because of the kind of health education I was given. I listened to the video that day, and that made me to be coming to this facility regularly during pregnancy” (*Service User in Kutunku, FCT*).

To reinforce the benefits of videos for service users, Mrs Buba (not her real name) from Ondo state narrated how watching videos during ANC classes deepened her understanding of self-care and nutritional requirements during pregnancy. According to Mrs Buba: “*When we come here on clinic days and after we finish singing and exercising, the nurses will tell us to watch videos of pregnant women, to see the type of food they prepared for them and how pregnant women gave themselves plenty of rest. Or if they are having pains in their tummy, how they rushed down to the clinic to see the nurse*.” Mrs. Buba also recounted how learning about food hygiene and better eating habits during pregnancy helped her to adopt better eating behavior. Mrs. Buba went on to have a successful delivery and brought her 6-month-old baby to the health facility for regular immunization.

##### Convenience of Offline Videos as Aide-mémoire for Clinical Practice

Linked to using digital technology for improving healthcare delivery, once the eLearning videos were downloaded from the VTR Mobile platform onto tablet computers, participants were able to watch the training content repeatedly at no additional cost. The convenience of offline access to clinical videos stimulated habitual use of clinical videos as reference material to guide live-saving procedures.

“I think some staff are beginning to look at this thing [clinical videos on tablets] as an important, err, aspect of work. The video helps us…it makes the work easier. If we are in any difficulty, we just go to that particular video and watch it many times. And now, we know what to do for clients” (*Facility manager in Gwagwalada, FCT*).

This extract demonstrates frontline health workers' reliance on clinical videos for increasing understanding and for problem solving. Staff met in groups to watch clinical videos on tablet computers as video viewing sessions enhanced knowledge exchange, peer support and troubleshooting.

#### Three External Barriers to the Uptake and Use of Digital Technology

Despite widespread positivity toward VTR and data digitization interventions, three external (structural) barriers affected the use of these technologies: (i) workload issues associated with technology introduction, (ii) poor internet connection, and (iii) poor electricity supply.

##### Dimensions of Workload Issues Associated With Technology Introduction

Two aspects of workload issues were identified from analysis of interview transcripts: (i) Lack of time to watch all clinical videos on the VTR platform, and (ii) workload associated with maintaining two health data registers—hardcopy registers and data entry into the data digitization App.

While the VTR intervention provided 40–60 h of video training to ensure FHW access to reliable MNCH information, however, most health workers interviewed in Ondo and the Kano states reported difficulty with finding time to watch all VTR clinical videos in the workplace due to increasing workload and tight scheduled associated with the introduction of the eHealth project. State policymakers and the local technology company addressed workload issues by providing alternative access to the VTR Mobile App, e.g., installing the App on personal mobile phones of FHWs to facilitate self-study at home.

“[For staff struggling to access the videos] … they [local technology company] have made it so easy for them to upload VTR on their phones [of FHWs]. During the pilot study, we encouraged them [FHWs] to get it. Those who have android phones can have it on their phones, so that it is not only when they get to the health facility where they have only one tablet that they can watch the videos” (*Policymaker in Ondo state*).

Regarding the second dimension of workload issues, FHWs in Ondo and Kano States cited increasing workload associated with entering data into the data management App, as well as maintaining hardcopy/paper registers and patient records cards in health facilities. A typical comment about experiences of using the data digitization App is:

“Yes, it [i.e., data management App] helps although it has added to our work in the sense that, before [introducing the App], we were filling only hardcopy registers. So, it will better, if they [policymakers] stopped the use of the registers and gave us [health staff] tablets for data collection and submission. But we are expected to first fill the registers, and then open tablet and input data from the register to the tablet one after the other. So, it has added to our work” (*Facility Manager, Onikokodiya, Ondo State*).

While this facility manager thought that the workload issues could be resolved by stopping the use of hard copy registers, conversely, the State Ministry of Health opted to train more FHWs in health facilities to use the data digitization App rather than stop the use of paper registers. It is unclear whether State MOH have trained more FHWs to use the technology.

##### Internet Connectivity and Electricity Supply

A few participants in health facilities served by 3G mobile networks reported difficulty with accessing both the VTR and data digitization Apps due to poor internet connectivity and poor electricity to power computer tablets. The narratives below from FHWs capture this sentiment:

“Data entry and transmission from the App goes fast except for days when there is no internet connectivity. We've actually been experiencing that for the past 2 months… those are the periods we actually have down times but aside from that, it's been okay. There are times when I have to turn on my private Wifi, but if I don't have data, then we have to wait till other members of the team come around and then they help us send data to the LGA” (*FHW in Gwagwalada, FCT*).

“Patients were happy, and they were patronizing this facility. The only problem we have is that we don't have electricity in the facility to power the tablets to watch videos. Secondly, at night, there is no security man here, so we can't stay back to take deliveries. You know, due to the security issue…we can't. Only one person cannot stay here [at night without electricity]” (*FHW in Gwagwalada, FCT*).

It is important to mention that internet access was not influenced by affordability issues as tablet computers in health facilities were loaded with pre-paid data plans to enable seamless access to both Apps. The first quote above indicates that during periods of poor internet connectivity, FHWs sometimes used their personal pre-paid data plans to transmit organizational data from tablet computers in facilities to the LGA headquarters. Regarding electricity supply, the second quote illustrates the inability of FHWs to charge devices when the rechargeable solar batteries installed at participating health facilities were drained/flat. The quote also introduces an alternative dimension of the lack of electricity. Unavailability of electricity at night made the largely female FHWs concerned about their personal safety, thereby preventing FHWs from providing needed services to service users (36).

Taken together, the foregoing six drivers/facilitators of acceptance and three barriers to use of new technology broadly affected the effectiveness of digital health interventions in Nigeria. Regardless of barriers to technology use, stakeholders reflected views that VTR and data digitization Apps are promising tools for: (a) providing high-quality and reliable training, (b) enhancing FHWs skills, (c) improving data management, and (d) increasing service uptake by pregnant women in Nigeria.

The next section assesses the extent to which the central program theory outlined in the Theory of Change (Figure 1) was borne out after 2 years (March 2017–2019) of project implementation. The central proposition of the eHealth project was that: “Participation of Frontline Health Workers, combined with health system interventions (VTR, digitization of data and availability of satellite communication infrastructure) deployed within a favorable environment at individual, organizational, and system levels, will improve standards of healthcare provision, utilization of MNCH services and health outcomes in Nigeria.

We will highlight the extent to which implementation of health systems interventions (of SatCom, VTR and data digitization technologies) generated project outcomes and impacts, and which other factors influenced the outcomes and impact. Five outcomes outlined in the project's ToC: (i) staff motivation and satisfaction, (ii) increased staff confidence to perform healthcare roles, (iii) improved standards of care leading to increased attendance at PHC facilities and utilization of MNCH services, (iv) greater use of accurate and reliable data for health systems management and decision-making, and (v) increased adoption of eHealth innovations in health and non-health sectors. We drew on findings of the legacy evaluation (conducted 12 months after project closedown) compared against findings of the end-line, midline and/or baseline evaluations to assess the (non-)achievement of project outcomes.

#### Effects, Outcomes, and Impact of Digital Interventions

##### Effectiveness of Deploying SatCom Technology

Findings of the midline and end-line evaluations demonstrated the viability of using Satcom technology to extend healthcare services to technologically disadvantaged regions of Nigeria. Over the 2-year period of the project, SatCom provided uninterrupted connectivity that enabled simultaneous and sustained deployment of VTR and data digitization in 75 facilities (about 60%) of 126 health facilities in the FCT, Kano, and Ondo states that lacked access to existing 3G mobile network. Without SatCom, the delivery of these interventions would be unattainable in health facilities located in rural areas.

Notwithstanding the success of the SatCom approach, findings of the legacy assessment (37) revealed that the SatCom infrastructure was disabled in 40 health facilities (37 in Ondo and 3 in the FCT) after the closedown of the project in March 2019. From September 2019 onwards SatCom technology was replaced by *Very Small Aperture Terminals* (VSATs) technology in health facilities that lacked access to 3G mobile network. This entailed deploying a VSAT at a central location to serve multiple health facilities over a wide geographic area, with a plan for State Governments to scale up the VSAT solution. This plan was however disrupted by the COVID-19 pandemic. The disruption has implications for the sustainability of results of the eHealth project given budget pressures on state governments for managing the COVID pandemic.

##### Availability of Accurate and Reliable Data to Support Decision-Making

Evidence from the midline and end-line assessments (38, 39) showed that implementing SatCom and data digitization interventions substantially increased the capacity of intervention health facilities to generate and transmit timely and accurate data via the InStrat server to health planners and policymakers, in summary form, to support policymaking at LGA and state levels.

Unexpected findings of this study were that, compared to their peers in the FCT and Kano State, facility heads in Ondo State leveraged the availability of reliable and accurate data at facility-level to inform: (i) disease surveillance and health promotion activities, and (ii) human resource management. While we had expected that policymakers would use health data to inform decision-making, we had not foreseen facility-heads using the digitization App in such ways. The quote below explains the process of using facility-level data for decision-making:

“If we collect data on malaria [using this App] and it shows a high rate of malaria, then we consider what we can do, e.g., give health education or distribute bed nets and go out to the community and tell the people that during the rainy season, they should dispose of all containers that may gather water and mosquitoes. Then, if there is an outbreak at the end of the month and maybe we never used to see more than 5–6 malaria cases on our register, and we discover [from computer tablet] that it has increased to about 13 cases, we will act based on what we are seeing. So, we now take measures to prevent more spread in the community” (*Head of Facility in Ondo State*).

The remarkable finding about facility heads in Ondo state was that besides using the data digitization App to transmit healthcare data, they adapted the App for transmitting urgent administrative and human resource information to the LGA headquarters. For instance, they used the App to apply for short-term leave for FHWs.

However, accessibility to the data digitization App and/or use available data for facility-level decision making was interrupted from June 2019 onwards, after project funding was withdrawn. The withdrawal of funding also affected electricity supply to power the tablets (which had been provided by solar panels during the project) and internet connectivity (previously enabled through SatCom) to transmit data to LGAs. Constrained access to the data digitization App has caused most health facilities to revert to manual data management using paper and hardcopy registers for data capture and reporting. The quote below highlights this sentiment:

“…We cannot send [data] with the iPad anymore, that is it. You know, the advantage of using the data digitization App far outweighs the burden that comes with using it…Ha, we now have to carry those registers sir, it is the ‘BAGCO bag’ that we are using to carry them o (laughs) you know. And we carry many registers, including the ANC register that is smaller, all these ones they are very bigger registers. So, it is not easy for us to be carrying all these bags to LGAs for verification” (*Facility Head, in Ondo State*).

##### Sustained Staff Motivation and Confidence

Findings of the legacy evaluation (37) suggest that accessibility to eLearning training content and clinical videos were neither affected by the discontinuation of SatCom infrastructure nor the withdrawal of project funding. Testimonies from FHWs identify the VTR intervention as the most significant change seen in the 12 months following project closedown. They explained how continuing access to clinical videos has sustained FHWs' knowledge and skills, and boosted their self-esteem, motivation and confidence to manage pregnancies, monitor women in labor and prevent deaths in babies:

“It [i.e., clinical videos] has certainly affected the level of staff confidence, I will like to use myself as an example. As nurse-midwife and most of these things [about maternity care] were obvious to me…but having watched them again in the videos, when I ask questions to clients now, I ask them as a confident professional, I don't ask like a novice anymore. It has increased my confidence. It has affected my motivation strongly, because each day I report to work, I want to learn new things and there is always something, [a topic] to watch, and it's motivated me to want to do something., I now tend to assist in other things when help is needed” (*Frontline Health Worker in Gwagwalada, FCT*).

The above stories of the sustained impact of VTR on staff motivation and confidence to perform clinical roles and take on new tasks have implications for the standard of MNCH services.

##### Longevity of Improvement in Standard of Care With Increased Patient Attendance

In narrating the most significant changes they experienced over the lifetime of the project and beyond, FHWs and facility heads across the three states cited sustained improvements in the standard of healthcare provision following the introduction of the VTR intervention in 2017. In the stories below, a facility head combines the themes of improved monitoring of pregnant women during labor with improved neonatal care to corroborate the impact of VTR on clinical care. The stories detail: (i) how and when the facility manager learned vital life-saving skills from clinical videos, (ii) her decision to introduce group video sessions for training more junior staff, and (iii) how the process contributed to increased standards of healthcare in her health facility.

“I will start with, erm, the use of partograph. Before this project started, we were not using it [partograph]. Even if we picked up the partograph, it was so confusing to us. But when the eHealth project brought this tablet [loaded with videos], I learnt how to use the partograph through the tablet. We watched the video together and I stepped down what I learnt to my staff. I photocopied the partograph and we started the practical [use of the partograph] here, so now every staff taking delivery here knows when to use the partograph. So we know that, as we are charting [events on the partograph], it will be telling you when there are danger signs or to refer at the correct time so that you won't be late [in referring]. Even the LGA officials gave us “thumbs up” for using the partograph to save the lives of women.”“Second, is about helping babies to breathe well. When these videos were introduced, we watched them and saw what we were supposed to do [when using Ambu bags]. Now when a woman is on a delivery couch, my staff will be ready here with their sterile Ambu bag waiting in case newborn baby needs to be helped. So, this video has helped us. We have saved so many lives, of these very small babies… I am very happy because even if I am not around, my staff will do it correctly. So, we are now equipped to help these babies to breathe” (*Facility Head, Gwagwalada in FCT*).

These stories demonstrate the effectiveness of continuing engagement with clinical videos for heightening the skills, alertness, and staff capability to detect early signs of complications and prevent needless deaths among women and infants. An alternate dimension of improved standard of care is staff-patient relations, which is captured in the next extract:

“For me particularly, [I like] the friendliness of the staff, the way they talk to you, the way they receive you, they pet you when you are pregnant, or when you are in labor or when you are a breastfeeding mother. If you did something that was not correct, e.g., if you breastfeed your baby in a way that is not proper, they will correct you gently. They will show you like…this is where you are supposed to put the nipple to the mouth of your baby, or one of them will come [to you] and… [explain that] it has happened to them when they were pregnant. So that makes you realize that they care about you, you understand? I love that, it is encouraging” (*Service User in Zuba, FCT*).

Patients attributed the reported changes in healthcare delivery to the introduction of clinical videos in health facilities and their use for patient education at ANC classes, which subsequently shaped patient attendance and use of MNCH services (already reported in section Perceived Usefulness of Technology for Improving Individual Performance).

#### Increased Adoption of eHealth Innovations in the Health Sector

Despite the closedown of the project, evidence from the legacy evaluation suggests that the eHealth project has seemingly generated significant interest in government and from other stakeholders in the health sector to adopt the eHealth project approach. Key examples include: (i) Ondo State's adoption of digital technology to support the implementation of a ***new Contributory Health Insurance Scheme***. In a public-private partnership with InStrat, the Ondo State Government has (in December 2019) adopted a package of inter-related digital health innovations (including digitization of health data and an electronic health insurance platform) to facilitate equitable access to essential healthcare services as a pathway to achieving universal health coverage. This new project, currently implemented in nine general hospitals in the Ondo State, is being incrementally expanded to 203 PHC facilities in the state; (ii) partnership between InStrat and the multiple State Governments using an adaptation of the VTR Application to ***develop a COVID-19 training App*** to provide FHWs with accurate and up-to-date information about COVID-19 to enable them to quickly identify, screen and manage COVID-19 suspects across multiple states in Nigeria (34).

In summary, this section identified four tangible impacts of digital technology on micro, meso and macro levels of the health system in Nigeria. There is sufficient evidence from analysis of multiple datasets to demonstrate that sustained deployment VTR and data digitization interventions successfully: (i) increased staff motivation and satisfaction (micro level), (ii) increased staff confidence to perform healthcare roles (micro level), (iii) increased the standard of MNCH care (meso level), and (iv) generated interest among government agencies (policy level) to increase adoption of eHealth innovations in the health sector. Although SatCom technology originally provided uninterrupted connectivity that stimulated greater use of accurate and reliable data to support policymaking, nevertheless, this impact was reversed after the withdrawal of project funding in March 2019.

## Discussion

Digital health technologies are infrequently brought to scale despite widespread enthusiasm about their potential to strengthen health systems by broadening access, improving quality of healthcare provision and developing essential ICT infrastructure [30]. This study aimed to assess if and how using digital interventions to extend health services to remote areas of Nigeria improved the standard of maternal, newborn and child health (MNCH) services. Specific objectives were to: (i) identify contextual drivers of acceptability and use of video training and data digitization interventions deployed at scale in Nigeria, and (ii) assess the impacts of digital interventions on different levels of the health system.

Analysis identified two broad environmental factors that shaped adoption of technology at policy level in Nigeria (section Contextual Enablers of Technology Adoption by State Governments). First, a supportive policy environment at Federal Government level inspired the adoption in 2013, of ICTs as a priority strategy for saving the lives of vulnerable women and children in Nigeria, coupled with the implementation of state-level ICT policies in the three participating states of the project. Second, a thriving private-public partnership between a local technology company and State Governments provided both funding and technical expertise for developing and implementing digital interventions.

Using a modified technology acceptance model (mTAM) to guide data analysis we also identified six drivers of acceptance and use of digital technology by health workers. The six drivers of acceptance are: (i) perceived ease of use of technology, (ii) perceived usefulness of technology to enhance job performance, (iii) offline access to clinical videos, (iv) access to tablet computers in the workplace, (v) prior training to increase familiarity with technology, and vi) ongoing technical support to ensure the functionality of digital technology. The first two facilitators of acceptance support the tenets of classic technology acceptance models (TAMs) that regard perceived ease of use, and perceived usefulness of technology as the key drivers of acceptance (26), while the latter three facilitators of acceptance help to extend the classic TAMs by acknowledging and incorporating the influence of contextual factors in shaping acceptance of new technology (40). Consistent with this, three contextual facilitators (or organizational support factors) that shaped the use of VTR and data digitization tools were prior training to prepare FHWs to embrace technology, access to technology in health facilities, and availability of technical support throughout the eHealth project. On the other hand, three contextual barriers that inhibited the use and effectiveness of digital technology in Nigeria were: (i) increasing workload following technology introduction that prevented FHWs from watching all clinical videos on the VTR platform, (ii) poor internet connection, and (iii) poor electricity supply that prevented access to the digital platform and re-charging of tablet devices in in rural areas.

Regarding impacts, findings within this study identified four tangible impacts of digital technology on micro, meso and macro levels of the health system in Nigeria (sections Effectiveness of Deploying SatCom Technology, Availability of Accurate and Reliable Data to Support Decision-Making Sustained Staff Motivation and Confidence, and Longevity of Improvement in Standard of care With Increased Patient Attendance). The impact of sustained deployment of multiple technologies (SatCom, video training and data digitization interventions) over a 2-year period on the health workforce building block of the health system highlighted the pivotal role of uninterrupted/offline access and reference to clinical videos for increasing FHW knowledge, that then generated increased motivation and confidence (individual level) to perform healthcare roles which together led to improved clinical skills. For the service delivery building block, better-trained and skillful FHWs felt empowered to provide high-quality MNCH services, that manifested as improved staff-patient relations, improved client engagement during ANC classes that led to improved ANC attendance, early detection of high-risk pregnancies and management of birth complications (institutional level). This emphasizes the significance of organizational readiness to provide essential material and human resources for achieving the delivery of high-quality care, even though this is not always present in LMICs [40]. Finally, ensuring uninterrupted connectivity via the combined SatCom and 3G mobile network approach during the project period strengthened information and data management systems that inspired trust and greater use of accurate and reliable data to influence policy decisions and governance (macro level), which manifested as increased adoption of eHealth innovations in the health sector. Unfortunately, some of the tangible benefits of SatCom and data digitization interventions were gradually reversed after withdrawal of project funding in the second quarter of 2019.

### Comparison With Other Studies

The findings of this study align with previous studies that implemented digital technology at scale in Nigeria and other LMICs, which found that: (i) widespread support for computer-enabled eLearning and data management, (ii) prior training to prepare workers to increase familiarity with technology improved usability, (iii) digital innovations were effective for enhancing workers' skills, attitudes and confidence, and client uptake of MNCH services, and (iv) health workers felt empowered in situations where technology boosted their needs and improved service delivery (16, 41, 42). These findings have implications for implementing strategies that ensure adequate orientation and continuing technical support for FHWs to adopt and utilize digital technology to achieve both individual and organizational goals. Furthermore, these findings increase the evidence base of the effectiveness of digital technology for improving the standard of MNCH services in LMICs [4].

While many studies focused on the efficacy of single ICT strategies for improving single health service problems, e.g., sending reminders to women to attend ANC appointments, and/or having children return for vaccinations (43, 44), our study assessed the effectiveness of implementing multiple digital interventions to address multiple health systems problems (limited access to training opportunities, deficient data management, and poor service delivery). Given the breadth of health systems building blocks impacted by digital health tools deployed in the eHealth project in Nigeria (SatCom to provide connectivity, VTR to train health workforce, and data management tools to improve information and data systems), our findings provide confidence to support the value of simultaneous implementation of multiple digital health tools for strengthening the health system in LMICs.

Similar to studies in Ghana and Indonesia (45, 46) we found that adequate training and simplicity of functionality of technology empowered the health workers to use technology irrespective of their level of education. Training often includes, but not limited to, introducing FHWs to how digital devices function, visual instructions for using new technology, and technical support for addressing problems. Besides improving usability, evidence from Ethiopia and South Africa indicate that sufficient prior training and regular technical support, for e.g., digitizing registries in MNCH improve the timeliness of data collection, reduce error rates, and increases data completeness (47, 48).

Despite growing recognition that technology use can improve FHWs' motivation and performance (41, 49), few studies have explained the underlying reasoning (50) for how technology interacts with FHWs, health systems and the implementation context to shape FHW motivation, performance, and quality of MNCH care (42). Our study identified five mechanisms by which digital technology generated impact which showed that access to tablet devices that were simple to use, and continuous engagement with clinical videos, led to increased knowledge, based on which FHWs: (i) felt confident, better trained and (ii) motivated to do their work. Better trained FHWs, in turn, (iii) felt supported with material resources, and (iv) empowered with enhanced skills that generated improved performance (individually and collectively) and ultimately improved MNCH services provision within institutional contexts of manageable workload, uninterrupted internet access, and electricity supply. Finally, improved data management systems inspired: (v) trust and greater use of accurate and reliable data for policy decisions and governance which led to increased adoption of eHealth innovations in the health sector.

### Study Strengths and Limitations

Three distinctive features of this study are its identification of: (i) contextual enablers of technology adoption at policy level by State Governments in Nigeria, (ii) drivers of and barriers to acceptance and use of technology by health workers, and (iii) mechanisms of impact of digital technology on micro, meso, and macro levels of the health system.

There are two limitations of the study. First, although it identified plausible mechanisms of impact of deploying multiple digital technologies at the individual, organizational and policy levels, this paper excludes a comparison of factors (individual, technology-related, and contextual factors) that motivated technology use among different stakeholders—CHEWs, nurses, and facility managers. This would have unraveled similarities and differences in motivations for using technology by different stakeholders, including for example, how years of experience of FHWs shaped technology use among stakeholders. Second, the study findings are based on FHWs self-reported responses drawn from semi-structured interviews conducted over four time periods (baseline, midline, end-line, and 12 months after project closedown). As the study adopted a qualitative research design, we did not include psychological assessments of the potential interactions between different mechanisms of impact of identified and their combined effects on performance.

## Conclusions

The range of interventions implemented by the eHealth project in Nigeria led to tangible and reported benefits across all stakeholder groups. This study demonstrates that simultaneous and sustained implementation of multiple digital technologies at scale enabled via SatCom and 3G mobile networks are viable approaches for strengthening multiple health systems building blocks (in this case, human resources, service delivery, information systems, and governance) to achieve the overall goals of the health system that includes improving the health and wellbeing of people of ages. Our findings extend the evidence base for the effectiveness of digital health technologies and for theoretical underpinnings to guide further technology use to support improvements in MNCH services in low resource settings.

## Data Availability Statement

The original contributions presented in the study are included in the article/Supplementary Material, further inquiries can be directed to the corresponding author/s.

## Ethics Statement

Ethical approval for the study was granted by the University of Leeds, School of Medicine Research Ethics Committee (MREC16-178), the Ondo State Government Ministry of Health (AD.4693 Vol. II/109), the Kano State Ministry of Health (MOH/Off/797/T1/350), and the Federal Capital Health Research Ethics Committee (FHREC/2017/01/42/12-05-17). The patients/participants provided their written informed consent to participate in this study.

## Author Contributions

BE and BO jointly conceived the study. BE and MJA developed the manuscript and led the writing of this paper with contributions from BO, GOA, AA, JT, KO, DA, OD, RY, OO, and MKA. All authors read and approved the final version of the manuscript.

## Conflict of Interest

OO is Co-Founder and CEO of InStrat Global Health Solutions, the company that implemented the digital technologies used in the outlined study. The remaining authors declare that the research was conducted in the absence of any commercial or financial relationships that could be construed as a potential conflict of interest.
