# Supplementary material for: What Are the Contextual Enablers and Impacts of Using Digital Technology to Extend Maternal and Child Health Services to Rural Areas? Findings of a Qualitative Study From Nigeria
Source: Front Glob Womens Health. 2021 May 14;2:670494. doi: 10.3389/fgwh.2021.670494 (PMC8594044; doi:10.3389/fgwh.2021.670494)
Supplement: Supplementary file 2 [file Data_Sheet_2.PDF]

Supplementary Material 2: Interview guides

Evaluating impact of Extending Health Services to remote areas of Nigeria using SatCom

Midline Assessment

In-depth Interview Guide for Heads of Health Facilities and Policy makers

Guidance notes

Before the interview

- Specify how long IDIs will take. You should familiarize yourself with the question guide and mark priority questions for the interview on the interview guide accordingly.
- You should also be familiar with the definitions and the terminology used in the project glossary (if one exists). You should be ready to clarify certain concepts and what they mean to the respondents during the interview
- As the interviews will be audio-recorded, you should check that the recorder is working properly before going into the interview.
- Check that you have notebooks and pens for note-taking and/or laptop computers as appropriate.
- Take extra copies participant information sheet, informed consent and other key documents that are deemed useful for each interview.

At the beginning of an interview

- Introduce yourself and other researchers present (if applicable) to the respondent.
- Briefly explain the objectives of the research and obtain informed consent.
- Check the overall time available for the interview.
- Explain that this will be an open interview and ascertain that respondent understand what is expected of them e.g. "In your own words, we would like you to tell us your personal knowledge and experiences of providing oversight within the health systems in Nigeria."

During the interview

- Although we are interested in the respondent's own views and experiences, for the purpose of the interviews, you are allowed to tactfully interrupt respondents to clarify issues related to the initial working theories or provide prompts. But do not try to always fill the silence with a next question or probe—your respondent may need some time to think about the issue.
- The bullet points in the next page provide a checklist of possible issues to raise. Use your judgement and ask these bullet points only if the conversation is faltering, or if the respondent is deviating too much from the question you asked them. Please note that some bullet points may not be applicable to some respondents.
- Keep track of the questions asked;
- Take note of non-verbal clues that might be important for analysis.
- If the interview is not being recorded (due to respondent refusal, or equipment failure), the note-taker's priority is to summarise the content of the interview
- During the interview, the interviewer should listen and be prepared to ask follow-up questions that further clarify issues.
- Use probes carefully; you do not need to ask all probes for all questions. Use your judgement and apply probes when the respondent is not answering fully or freely, or if the respondent doesn't appear to understand the question.

- Probe for specific examples from respondent's experience in support of her/his statements where possible.
- If respondent is not answering a question in detail, ask 'give-me-an-example' type question to complement the question route.
- Always let the respondent finish a phrase; try not to interrupt her/him
- Use the interview guide flexibly, allowing the respondent to address the questions in any order but ensuring that the key issues are covered.

After the interview

- Thank the respondent/interviewee for their participation
- Remember to catalogue the tape/file (if you are recording)
- During debriefing, reflect on, discuss in the research team and clearly note down any other issues (e.g. respondent's mood during the pauses, your general impressions of the interview, good questions to ask) that may be important for future interviews, and the data analysis.
- Transcription if possible should start the day of the interview or at most the next day.

**Information area**	**Example questions and probes**	***Comments***
**Introduction by respondent**		
	For the tape, can you introduce yourself, and describe your work/what you do?	This question is to get respondents talking
**Section A:**<br><br>**Staff training**	1. Before the use of video training in this facility (or LGA), how were your staff trained?	Historical insight into training practice in LGA or health facility
	2. Before the use of video training in this facility (or LGA), what was the regularity of staff training (monthly, yearly, 2-yearly etc.) and why? please give reasons for your answer<br><br>**Probe:** Categories of staff that benefited from regular training and why?<br>• Categories of staff that were left out and why?<br>• How trainings were conducted (logistics, for example<br>• Where the trainings were conducted?<br>• Who or which organization provided the trainings and why?	
	3. In your opinion, how has using video training for staff training in this facility (or LGA) compared with using face-to-face training, meetings, workshops, seminars etc.?<br>**Probe:** How did the adoption of video training affect:<br>• The duration or timing of training programmes<br>• Availability of staff at their duty posts during training<br>• Financial reward for attending trainings, workshops and seminar.	Comparison of e-Health application to other modes of training
	4. What difficulties or challenges did **you, anyone** or **your facility** face regarding using video training to train health workers in this facility (or LGA)?<br><br>**Probes:**<br>• Did challenge(s) mentioned above affect standard of healthcare delivery in your facility? If yes, how?<br>• How was the challenge addressed or overcome?	Challenges/ difficulties experienced with using VTR to train staff

Information area	Example questions and probes	*Comments*
	5. In your opinion, how did the use of video training affect how health staff are trained in this facility (or LGA)? please give reasons for your answers<br><br>***Probe:*** Both positive and negative response are Ok here.	Effect of e-Health application on staff training
**Section B:**<br><br>**Data management**	6. In your opinion, how has the use of CliniPAK application affected how data is collected and managed in this facility (or LGA).<br><br>**Probe:** Both positive and negative responses are Ok here.	Effect of e-Health App on data collection and managed
	7. How has the use of CliniPAK application affected who/which department/unit is responsible for collecting and/or analyzing the data in this facility?<br><br>**Probe:** Has this changed? If yes, how?	Effect of e-Health App on responsibility for collecting and analyzing data
	8. How has the use of CliniPAK application affected the way health services data is transmitted and reported to the LGA headquarters?<br><br>**Probe:** Has this changed? If yes, how?	Effect of e-Health App on data transmission and reporting
	**9.** How has the introduction of CliniPAK application affected the way health service data is validated by the LGA headquarters?<br><br>**Probe:** Has the way of validating data changed? If yes, how?	Effect of e-Health App on data validation
	10. How has the introduction of CliniPAK Application affected who/ which department/unit was responsible for transmitting and/or reporting the data in this facility?<br><br>**Probe:** Has this changed? If yes, how?	Effect of e-Health App responsibility for data transmission and reporting
**Section C:**<br><br>**Supervision**	11. In this facility, are FHWs supervised? If yes, how are they currently supervised? Who supervises them? And why are they supervised?<br><br>**Probe:** The frequency of supervision (daily, weekly, monthly, yearly etc.) and why? please give reasons for your answer	Insight into practice of supervision
	12. What difficulties or challenges did you face regarding supervision of health workers?<br><br>**Probes:**<br>• Did challenge(s) mentioned above affect standard of healthcare delivery in your facility? If yes, how?<br>• How was the challenge addressed or overcome?	
	13. What recommendations do you give to improve how supervision is done in this health facility?	
**Section D:**<br><br>**Decision-making**	14. Since the introduction of CliniPAK in this facility/LGA, have you used eHealth data available from CliniPAK for decision-making in your health facility/LGA?<br><br>**Probe:**<br>• Details about decisions that were informed by CliniPAK data<br>• Has the implementation of CliniPAK influenced decision to adopt eHealth tools in this LGA/State?	

Information area	Example questions and probes	*Comments*
**Section E:**<br><br>**Existing policies and programmes**	15. From your experience, do you know of any policy or other projects in the LGA that promote or use e-health solutions for training FLHWs and or manage data?<br><br>**Probe:**<br>• Details about these policies or projects.<br>• Year the policy/projects started<br>• How the policy/projects is being implemented<br>• The performance of such policy or projects	
	16. Since the introduction of the VTR and CliniPAK in this LGA/state, has there been any policy document introduced or policy change in terms of using e-health solutions for health care delivery? If yes, give details.<br>**Probe:**<br>• When the policy document or policy change was introduced?<br>• What informed such policy document/change?	
	17. Since the introduction of the VTR project in this state, has there been expansion of use of VTR or CliniPAK to other health facilities (or LGAs) that you are aware of? If yes, give details.<br>**Probe:**<br>• When did the expansion to other facilities or LGA take place?<br>• What informed such expansion of use of VTR and/or CliniPAK?<br>• Do you know who/what organization funded the expansion?	
**For Policymakers Only**	**General questions related to IPP Nigeria evaluation questions**<br><br>**Relevance of project:**<br>• How has the project affected the standard of healthcare in the State /LGA?<br>• How are the project objectives related to State ICT and MNCH priorities?<br><br>**Effectiveness of project:**<br>• How has the project affected the health of women and their infants in this State/LGAs and communities?<br><br>**Efficiency of project:**<br>• How has the project affected government's allocation of resources to FHWs training and data management?<br><br>**Impact of project:**<br>• What practical difference is the project making to health and healthcare in affected LGAs or communities?<br>• What are positive and negative impacts of the project?<br>What are the primary and secondary long-term effects by the project, directly or indirectly, intended or unintended?<br><br>**Sustainability**<br>• How easy will it be for the LGAs and the state to take over project when donor input finishes e.g. from Inmarsat (SatCom)?	

Midline Assessment

In-Depth Interview Guide for Frontline Health Workers

Guidance notes

Before the interview

- Specify how long IDIs will take. You should familiarize yourself with the question guide and mark priority questions for the interview on the interview guide accordingly.
- You should also be familiar with the definitions and the terminology used in the project glossary (if one exists). You should be ready to clarify certain concepts and what they mean to the respondents during the interview
- As the interviews will be audio-recorded, you should check that the recorder is working properly before going into the interview.
- Check that you have notebooks and pens for note-taking and/or laptop computers as appropriate.
- Take extra copies participant information sheet, informed consent and other key documents that are deemed useful for each interview.

At the beginning of an interview

- Introduce yourself and other researchers present (if applicable) to the respondent.
- Briefly explain the objectives of the research and obtain informed consent.
- Check the overall time available for the interview.
- Explain that this will be an open interview and ascertain that respondent understand what is expected of them e.g. "In your own words, we would like you to tell us your personal knowledge and experiences of working within the health systems in Nigeria."

During the interview

- Although we are interested in the respondent's own views and experiences, for the purpose of the interviews, you are allowed to tactfully interrupt respondents to clarify issues related to the initial working theories or provide prompts. But do not try to always fill the silence with a next question or probe—your respondent may need some time to think about the issue.
- The bullet points in the next page provide a checklist of possible issues to raise. Use your judgement and ask these bullet points only if the conversation is faltering, or if the respondent is deviating too much from the question you asked them. Please note that some bullet points may not be applicable to some respondents.
- Keep track of the questions asked;
- Take note of non-verbal clues that might be important for analysis.
- If the interview is not being recorded (due to respondent refusal, or equipment failure), the note-taker's priority is to summarise the content of the interview
- During the interview, the interviewer should listen and be prepared to ask follow-up questions that further clarify issues.

- Use probes carefully; you do not need to ask all probes for all questions. Use your judgement and apply probes when the respondent is not answering fully or freely, or if the respondent doesn't appear to understand the question.
- Probe for specific examples from respondent's experience in support of her/his statements where possible.
- If the respondent is not answering a question in detail, ask 'give-me-an-example' type question to complement the question route.
- Always let the respondent finish a phrase; try not to interrupt her/him

- Use the interview guide flexibly, allowing the respondent to address the questions in any order but ensuring that the key issues are covered.

After the interview

- Thank the respondent/interviewee for their participation
- Remember to catalogue the tape/file (if you are recording)
- During debriefing, reflect on, discuss in the research team and clearly note down any other issues (e.g. respondent's mood during the pauses, your general impressions of the interview, good questions to ask) that may be important for future interviews, and the data analysis.
- Transcription if possible should start the day of the interview or at most the next day.

**Information area**	**Example questions and probes**	***Comments***
**Introduction by respondent**		
	For the tape, can you introduce yourself, and describe your work/what you do in this facility (CHEW, mid-wife, nurse, doctor etc.)?<br><br>**Probe:** Length of time staff has worked in this facility (in years)	This question is to get respondents talking
**Section A:**<br><br>**Staff training**	1. Before the introduction of video training in this facility how were staff trained?	Historical insight into training practice in health facility
	2. Before the introduction of video training in this facility, what was the regularity of staff training (monthly, yearly, 2-yearly etc.) and why? please give reasons for your answer<br><br>**Probe:** Categories of staff that benefited from regular training and why?<br>• Categories of staff that were left out and why?<br>• How trainings were conducted (logistics, for example)<br>• Where the trainings were conducted?<br>• Who or which organization provided the trainings and why?	
	3. In your opinion, how has using video training to train staff in this facility compared with using face-to-face training, workshops, seminars etc.?<br>**Probe:** How did the adoption of video training affect:<br>• The duration or timing of training programmes<br>• Availability of staff at their duty posts during training<br>• Financial reward for attending trainings, workshops and seminar.	Comparison of e-Health application to other modes of training
	4. What difficulties or challenges did **you, anyone** or **your facility** face regarding using video training to train health workers in this facility (or LGA)?<br><br>**Probes:**<br>• Did challenge(s) mentioned above affect standard of healthcare delivery in your facility? If yes, how?<br>• How was the challenge addressed or overcome?	Challenges/ difficulties experienced with using VTR to train staff
	5. In your opinion, how did the use of video training affect how health staff are trained in this facility (or LGA)? please give reasons for your answers	Effect of e-Health application on staff training

**Information area**	**Example questions and probes**	***Comments***
	Probe: Both positive and negative response are Ok here.	
**Section B:**<br><br>**Benefits of VTR and CliniPAK to frontline health workers**	6. From your experience, has participating in VTR and/or using CliniPAK been beneficial to you? If yes, what benefits you refer to.<br><br>***Probe:*** *Benefits of VTR can include but not limited to*<br>• Improved understanding of MNCH subject matter<br>• Preparing FHWs for their role in healthcare delivery<br>• Exposing FHWs to high quality training content<br>• Increased knowledge and skills<br>• Helping FHWs to perform their tasks<br>• Improvements in health of women and their infants in this LGA<br>• Improvements in standard of healthcare provided in this facility?<br>• Other benefits?	Views of benefits of participation in VTR and using CliniPAK
	7. How has your exposure to VTR training and/or exposure to using CliniPAK affected your confidence and self-esteem in discharging their duties? Please explain why this is the case.<br><br>***Probe:***<br>• How has participating in VTR training affected your self-confidence? Please explain.<br>• How has participating in the VTR affected your motivation to work as a health worker?<br>• In your opinion, why has VTR affected you in this way? Please explain.	Effect of eHealth tools on self-confidence, self-esteem and staff motivation to work
	8. How has your exposure to VTR training and/or using CliniPAK affected your job satisfaction? Please explain why this is the case.<br><br>***Probe:***<br>• How has participating in VTR training and/or using CliniPAK affected job satisfaction? Please explain.<br>• Why has VTR and/or CliniPAK had this effect? Please explain.	Effect of eHealth tools on job satisfaction
	9. What can be done to improve use of VTR for health worker training?	Recommendations for improving VTR use
**Section C:**<br><br>**Data management**	10. In your opinion, how has the use of CliniPAK application affected how data is collected and managed in this facility?<br><br>**Probe:** Both positive and negative responses are Ok here.	Effect of e-Health App on data collection and managed
	11. How has the use of CliniPAK application affected who/which unit is responsible for collecting and/or analyzing the data in this facility?<br><br>**Probe:** Has this changed? If yes, how?	Effect of e-Health App on responsibility for collecting and analyzing data

Information area	Example questions and probes	*Comments*
	12. How has the use of CliniPAK application affected the way health services data is transmitted and reported to the LGA headquarters?<br><br>**Probe:** Has this changed? If yes, how?	Effect of e-Health App on data transmission and reporting
	13. How has the introduction of CliniPAK Application affected who/ which department/unit was responsible for transmitting and/or reporting the data in this facility?<br><br>**Probe:** Has this changed? If yes, how?	Effect of e-Health App responsibility for data transmission and reporting
	14. How has the use of CliniPAK application affected the way health service data is validated by the LGA headquarters?<br><br>**Probe:** Has the way of validating data changed? If yes, how?	Effect of e-Health App on data validation
**Section D:**<br><br>**Supervision**	15. Regarding staff supervision, have you been supervised in this health facility in the past 1 year? If yes, how often were you supervised? Who supervises you? And why do you think you are being supervised?<br><br>**Probe:**<br>• Frequency of supervision (daily, weekly, monthly, yearly etc.) and why? please give reasons for your answer<br>• What aspects of your work were supervised?<br>• Did supervision involve skills check? If yes, what skills were checked?	Experience of being supervised
	16. Do you derive benefits from being supervised? If yes, what benefits?<br><br>**Probes:**<br>• What benefits do you derive from being supervised?	Benefits of supervision to FHW
	17. What recommendations do you give to improve how supervision is done in this health facility?	

Evaluating impact of Extending Health Services to remote areas of Nigeria using SatCom

Mid-line assessment

In-Depth Interview Guide for service users of health facilities

Guidance notes

Before the interview

- Specify how long IDIs will take. You should familiarize yourself with the question guide and mark priority questions for the interview on the interview guide accordingly.
- You should also be familiar with the definitions and the terminology used in the project glossary (if one exists). You should be ready to clarify certain concepts and what they mean to the respondents during the interview
- As the interviews will be audio-recorded, you should check that the recorder is working properly before going into the interview.

- Check that you have notebooks and pens for note-taking and/or laptop computers as appropriate.
- Take extra copies participant information sheet, informed consent and other key documents that are deemed useful for each interview.

At the beginning of an interview

- Introduce yourself and other researchers present (if applicable) to the respondent.
- Briefly explain the objectives of the research and obtain informed consent.
- Check the overall time available for the interview.
- Explain that this will be an open interview and ascertain that respondent understand what is expected of them e.g. "In your own words, we would like you to tell us your personal knowledge and experiences of making use of healthcare in this state."

During the interview

- Although we are interested in the respondent's own views and experiences, for the purpose of the interviews, you are allowed to tactfully interrupt respondents to clarify issues related to the initial working theories or provide prompts. But do not try to always fill the silence with a next question or probe—your respondent may need some time to think about the issue.
- The bullet points in the next page provide a checklist of possible issues to raise. Use your judgement and ask these bullet points only if the conversation is faltering, or if the respondent is deviating too much from the question you asked them. Please note that some bullet points may not be applicable to some respondents.
- Keep track of the questions asked;
- Take note of non-verbal clues that might be important for analysis.
- If the interview is not being recorded (due to respondent refusal, or equipment failure), the note-taker's priority is to summarise the content of the interview
- During the interview, the interviewer should listen and be prepared to ask follow-up questions that further clarify issues.

- Use probes carefully; you do not need to ask all probes for all questions. Use your judgement and apply probes when the respondent is not answering fully or freely, or if the respondent doesn't appear to understand the question.
- Probe for specific examples from respondent's experience in support of her/his statements where possible.
- If the respondent is not answering a question in detail, ask 'give-me-an-example' type question to complement the question route.
- Always let the respondent finish a phrase; try not to interrupt her/him
- Use the interview guide flexibly, allowing the respondent to address the questions in any order but ensuring that the key issues are covered.

After the interview

- Thank the respondent/interviewee for their participation
- Remember to catalogue the tape/file (if you are recording)
- During debriefing, reflect on, discuss in the research team and clearly note down any other issues (e.g. respondent's mood during the pauses, your general impressions of the interview, good questions to ask) that may be important for future interviews, and the data analysis.
- Transcription if possible should start the day of the interview or at most the next day.

Information area	Example questions and probes	*Comments*
**Introduction by respondent**		
	For the tape, can you introduce yourself, and describe your work/what you do?	This question is to get respondents talking
**Section A:**<br><br>**Purpose of and attraction of client to utilize health facility**	1. Please describe the type of service you came to access in this health facility?<br><br>**Probes:**<br>• *Type of service:* Whether to receive antenatal care (when pregnant), postnatal care (after delivery) or for other reasons (please specify)<br>• *How long have you been using this health facility?* (in months or years)	
	2. What specific reason(s) made you chose to attend this particular facility? Please give reasons for your answer<br><br>**Probe:** Quality of care, nearness to client's home, attitude of health staff, cost of service, etc.	Motivation for using health facility
**Section B:**<br><br>**Client's perception of attitude of health workers and quality of care**	3. Please, can you describe the way health staff in this facility usually receive you when you come for health care?<br><br>**Probes:**<br>• Health staff attitude toward patients (Positive and negative responses can be put here)<br>• The friendliness of the health workers<br>• Since you last used this facility, what changes in staff attitude have you observed? (Please note: this can be both positive and negative)	
	4. Can you remember how long it took the staff to attend to you after you got to the facility?<br><br>**Probes:**<br>• Please estimate the length of time (in minutes or hours as the case may be)<br>• Since you last used this facility, what changes have you noticed in the length of time it takes staff to attend to you?	
	5. What is your opinion about the state of cleanliness of the health facility?<br>**Probe:**<br>• Since you last used this facility, have you observed changes in cleanliness of facility? If yes, describe changes observed	
	6. In the past 12 months, have you observed the use of any new device(s) by the health workers in this health facility?<br>**Probe:**<br>• A description of the device (**Probe for CliniPAK if not mentioned)**<br>• What the device is used for<br>• If device has impacted on the quality of care from HW	

Information area	Example questions and probes	Comments
	7. In your opinion, did the staff offer you competent professional care?<br><br>***Probe:*** *If client* was satisfied with the standard of care s/he received?<br>• Reason why client felt s/he received competent care<br>• If client was not satisfied with standard care, ask for reasons<br>• What can be done to improve the standard of care<br>• If client would like to use this facility next time, if yes, ask for reasons<br>• If client would like to recommend the facility to a friend or a relation	
	8. Did you receive any counselling for your health condition? If yes, how will you describe the way you were counselled about your health condition?	
	9. In your opinion, where all your questions and concerns addressed by health workers?	
**Section C:**<br><br>**Client's perception of ethics standards of health staff**	10. Can you describe the level privacy given to you when you attended at this facility?<br>**NB:** Privacy here means to be given individual private space (as much as possible) to talk about one's health. E.g. were you attended to alone by a health worker in place no other person knew what you were discussing? Were you attended to in an open area where you felt comfortable talking? Did you feel that other patients/clients were too close and may have heard what you discussed with your care giver?<br>**Probe:** *If client was* satisfied with level of privacy she usually received.<br>• If yes, please explain why<br>• If not, please explain why not?<br>• What can be done to improve patient privacy while receiving care	
	11. In your opinion, would you say that health workers usually keep your medical information private, that is not discussing openly among health workers each time you visit this facility?<br><br>**Probe:** If client was satisfied with the degree of confidentiality in handling her information<br>• If yes, please explain reason(s) for satisfaction<br>• If not, please explain why not?<br>• What can be done to improve patient privacy while receiving care?	
	12. Can you tell us if you have suffered any form of verbal, psychological or physical abuse while receiving care in the facility?<br><br>**Probe:**<br>• Type of abuse If any<br>• Effect of the abuse on client.	
	13. What recommendations do you give to improve the care provided by health workers in this health facility?	
