# Supplementary material for: What Are the Contextual Enablers and Impacts of Using Digital Technology to Extend Maternal and Child Health Services to Rural Areas? Findings of a Qualitative Study From Nigeria
Source: Front Glob Womens Health. 2021 May 14;2:670494. doi: 10.3389/fgwh.2021.670494 (PMC8594044; doi:10.3389/fgwh.2021.670494)
Supplement: Supplementary file 3 [file Data_Sheet_3.PDF]

Manuscript: **What are the contextual enablers and impacts of using digital technology to extend healthcare services to rural areas? Findings of a mixed-method evaluation from Nigeria.**

Consolidated criteria for reporting qualitative studies (COREQ): 32-item checklist

Developed from:
Tong A, Sainsbury P, Craig J. Consolidated criteria for reporting qualitative research (COREQ): a 32-item checklist for interviews and focus groups. *International Journal for Quality in Health Care*. 2007. Volume 19, Number 6: pp. 349 – 357

No. Item	Guide questions/description	Reported on Page #
**Domain 1: Research team and reflexivity**		
Personal Characteristics		
1. Inter viewer/facilitator	Which author/s conducted the interview or focus group?	Pages 7 and 8 (section 2.5 data collection and section 2.6 data analysis)
2. Credentials	What were the researcher's credentials? E.g. PhD, MD	Page 7 (section 2.5 data collection)
3. Occupation	What was their occupation at the time of the study?	Page 7 (section 2.5 data collection)
4. Gender	Was the researcher male or female?	Page 7 (section 2.5 data collection)
5. Experience and training	What experience or training did the researcher have?	Page 7 (section 2.5 data collection)
Relationship with participants		
6. Relationship established	Was a relationship established prior to study commencement?	Yes, inferred from page 7
7. Participant knowledge of the interviewer	What did the participants know about the researcher? e.g. personal goals, reasons for doing the research	Inferred from pages 7 and 8 (Data collection and analysis sections)
8. Interviewer characteristics	What characteristics were reported about the interviewer/facilitator? e.g. Bias, assumptions, reasons and interests in the research topic	Reason for conducting the research (see aim of study in page 3 of manuscript)

**Domain 2: study design**		
Theoretical framework		
9. Methodological orientation and Theory	What methodological orientation was stated to underpin the study? e.g. grounded theory, discourse analysis, ethnography, phenomenology, content analysis	Pages 5, 6 and 8 (section 2.2 conceptual frameworks, and sections 2.5 and 2.6 i.e. data collection and analysis).
Participant selection		
10. Sampling	How were participants selected? e.g. purposive, convenience, consecutive, snowball	Page 7 (section 2.5 data collection)
11. Method of approach	How were participants approached? e.g. face-to-face, telephone, mail, email	Face-to-face: Page 7 (section 2.3 study participants)
12. Sample size	How many participants were in the study?	Page 7 (section 2.5 data collection)
13. Non-participation	How many people refused to participate or dropped out? Reasons?	Page 7: not provided
Setting		
14. Setting of data collection	Where was the data collected? e.g. home, clinic, workplace	Page 8 (section 2.5 data collection)
15. Presence of non-participants	Was anyone else present besides the participants and researchers?	Not applicable
16. Description of sample	What are the important characteristics of the sample? e.g. demographic data, date	Page 9 (section 3.1 characteristics of stakeholders)
Data collection		
17. Interview guide	Were questions, prompts, guides provided by the authors? Was it pilot tested?	Additional file 2 and page 10
18. Repeat interviews	Were repeat interviews carried out? If yes, how many?	Yes. There were 4 phases of data collection (baseline, midline, end-line and legacy assessment) see Table 4 in section 3.1

19. Audio/visual recording	Did the research use audio or visual recording to collect the data?	Page 8 (section 2.5 data collection)
20. Field notes	Were field notes made during and/or after the interview or focus group?	Yes.
21. Duration	What was the duration of the interviews or focus group?	Page 8 (section 2.5 data collection)
22. Data saturation	Was data saturation discussed?	No, Not applicable
23. Transcripts returned	Were transcripts returned to participants for comment and/or correction?	No, Not applicable.
**Domain 3: analysis and findings**		
Data analysis		
24. Number of data coders	How many data coders coded the data?	Page 8 and 9 (section 2.6 data analysis)
25. Description of the coding tree	Did authors provide a description of the coding tree?	Page 8 and 9 (section 2.6 data analysis)
26. Derivation of themes	Were themes identified in advance or derived from the data?	Page 9 Mainly derived from the data
27. Software	What software, if applicable, was used to manage the data?	Page 8
28. Participant checking	Did participants provide feedback on the findings?	No
Reporting		
29. Quotations presented	Were participant quotations presented to illustrate the themes/findings? Was each quotation identified? e.g. participant number	Yes, Page 10 to 16
30. Data and findings consistent	Was there consistency between the data presented and the findings?	Yes, there was.<br>Page 10 to 16
31. Clarity of major themes	Were major themes clearly presented in the findings?	Yes. they were.<br>From page 9 to 13
32. Clarity of minor themes	Is there a description of diverse cases or discussion of minor themes?	Discussion of major and minor themes<br>From page 10 to 16
